# Effect of Hardness Distribution on Strength of Narrow-Gap Hot-Wire Laser-Welded Joint for High-Tensile Strength Steel

**DOI:** 10.3390/ma18020297

**Published:** 2025-01-10

**Authors:** Jukkapun Greebmalai, Kazuyuki Matsumoto, Keita Marumoto, Motomichi Yamamoto

**Affiliations:** Graduate School of Advanced Science and Engineering, Hiroshima University, Hiroshima 739-8527, Japan; d225295@hiroshima-u.ac.jp (J.G.);

**Keywords:** laser welding, hot wire, narrow gap, high-tensile strength steel, HAZ softening

## Abstract

Application of high-heat input welding on high-tensile strength steels causes deterioration of mechanical properties of the welded joint, due to softening and grain coarsening in the heat-affected zone (HAZ). In this study, low-heat input narrow-gap hot-wire laser welding was applied to 12 mm thick 780 MPa-class high-tensile strength steel plate. Conditions were optimized based on microstructural observations of joints produced at various welding speeds. Heat input was estimated from measured grain size. Evaluation of properties of joints welded at 0.5 m/min revealed sound toughness, tensile strength, and elongation. The effect of undermatched weld metal width on joint strength was analyzed using a finite element method. When the width of undermatched weld metal was 2.5 mm, the joint strength was 99% of the base metal strength; when it was 7.5 mm, the strength dropped to 95%. The effect of HAZ softening width on joint strength with even-matched weld metals was similarly analyzed, showing that even when the HAZ softening width was 2.0 mm, the joint strength was 98% of the base metal strength. The results of this study suggest that narrow-gap hot-wire laser welding can efficiently reduce heat input and the HAZ softening zone, thereby achieving both high strength and high toughness.

## 1. Introduction

High-efficiency welding can be achieved by minimizing the number of passes, although a lower number of welded passes requires a higher material deposition ratio and higher heat input. During high-heat input welding, the heat-affected zone (HAZ) of the welded joint experiences grain coarsening proportional to the thermal effects of the higher heat input, which leads to deterioration in the mechanical properties of the joint. Studies have reported negative impacts on high-tensile strength steels during the welding process due to this heat-induced behavior [1,2,3,4]. Reducing the heat input is considered an effective method for mitigating joint deterioration; however, a lower heat input may lead to a decrease in welding efficiency.

Narrow-gap welding is a method characterized by using a small groove width with a small bevel angle, resulting in reductions in the amounts of deposition material and heat input, thereby enabling high-efficiency welding [5,6]. In this process, gas metal arc welding (GMAW) and gas tungsten arc welding (GTAW) require the torch to access the interior of the groove near the welding area. This access limitation means that the groove width cannot be too narrow. In contrast, laser welding can effectively reach and weld within the confines of an extremely narrow groove [7,8,9,10,11], benefiting from its small beam profile. In terms of material deposition or wire insertion, the hot-wire method involves feeding an electrically heated wire into the molten pool during the welding process. With the external heat source heating the filler wire, less additional heat from the arc or laser is needed to completely melt it into the molten pool. This technique allows for a higher amount of filler deposition, facilitating high-efficiency welding. Moreover, recent years have seen ongoing studies on hot-wire narrow-gap laser welding aimed at enhancing the efficiency of narrow-gap welding while maintaining lower heat input [12,13,14,15,16].

This study combined narrow-gap laser welding and the hot-wire method, aimed at achieving low-heat input welding, which reduces the heat-induced microstructural impact on the HAZ. In addition, an attempt was made to control the strength (hardness) and toughness of the weld metal by using a softer wire compared to the strength of the base steel, making use of the large degree of freedom in wire selection of the hot-wire method and the large cooling rate of this welding method.

Low-heat input welding has a higher cooling rate than high-heat input conditions [17,18] and hardness of low-heat input weld metal increases [18], which could lead to increased cracking risk [19,20]. To prevent crack initiation, a stress-reduction method involving the selection of a low-strength filler wire was implemented in this study of hot-wire narrow-gap laser welding, particularly for the welding of thick high-strength plates [21,22]. This approach results in a lower strength of the weld metal than that of the base material, leading to undermatched strength and a consequent decrease in joint strength. Both undermatched weldment strength and HAZ softening can diminish the overall strength of the joint.

Hochhauser et al. [23] conducted strength evaluations on thermo-mechanically controlled process (TMCP) high-tensile steels with varying widths of the softening zone, revealing no difference in base-metal strength when the softening zone width was 0.25 times the plate thickness. Similarly, Rodrigues et al. [24] reported that there was no difference in base-metal strength when the softening zone width was less than one-third of the plate thickness in high-tensile steels. Therefore, with the application of plastic constraints, it is possible to achieve high joint efficiency, even when the joint has a softening region. When the narrow-gap concept is carried out using the hot-wire laser welding method, which leads to low heat input and a narrow width of the weld metal and HAZ, it will be possible to achieve a welded joint with sufficient strength or equivalent to that of the base material despite undermatched strength of the filler wire.

This study clarifies the effects of heat input conditions on the microstructure, occurrence of incomplete fusion, and hardness distribution of joints welded using this technique. Additionally, evaluations of impact absorption energy and tensile strength were conducted using joints welded under optimized conditions. Welded joints with varying weld metal and softening widths were assessed using finite element method (FEM) analysis. Results regarding joint efficiency relative to weld metal and softening width are presented.

## 2. Materials and Methods

### 2.1. Materials and Experimental Method

**Figure 1:**
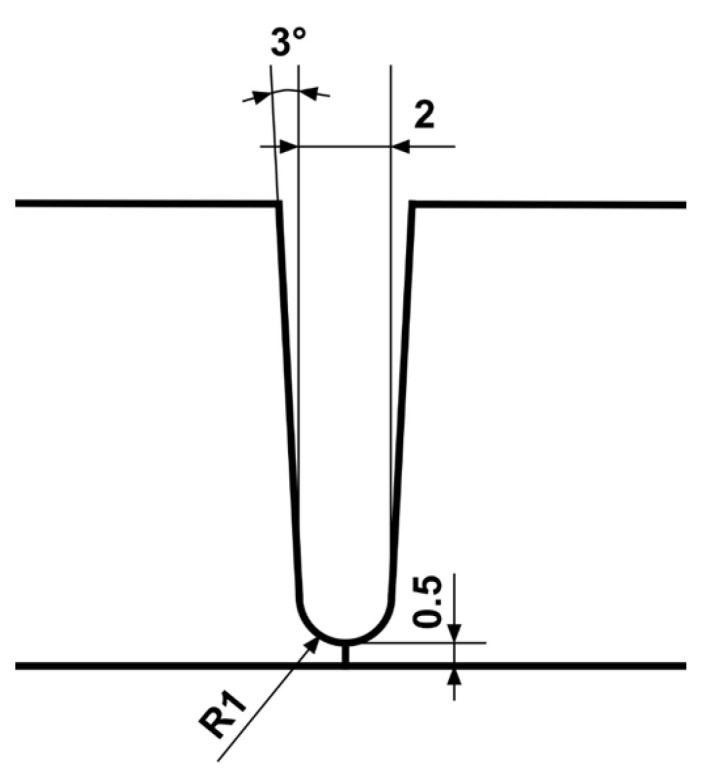
U-shaped narrow groove geometry (unit: mm).

In this study, 780 MPa-class high-tensile strength steels with plate thicknesses of 10 mm and 12 mm were used as the base metal. A narrow U-shaped groove was employed, with a 0.5 mm groove face and 1 mm radius at the bottom. The groove shape is shown in Figure 1. A 490 MPa-grade wire with 1.2 mm diameter was used as the filler material. Chemical compositions of the materials are shown in Table 1.

**Figure 2:**
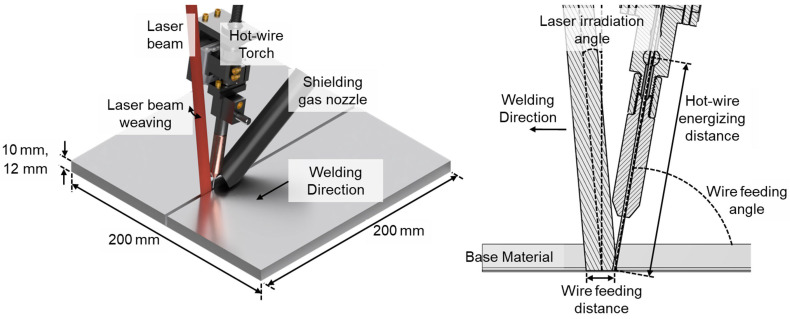
Experimental setup.

The experimental setup is shown in Figure 2. Welding was performed with the laser beam leading, and the filler wire and shielding nozzle were installed behind the molten pool. A rectangular laser beam of 1.6 mm × 11.0 mm with a focusing and homogenizer lens configuration was used. The filler wire tip was located at the rear of the laser beam spot at 10 mm from the front of the beam profile. Argon was used as shielding gas following the hot-wire torch.

**Figure 3:**
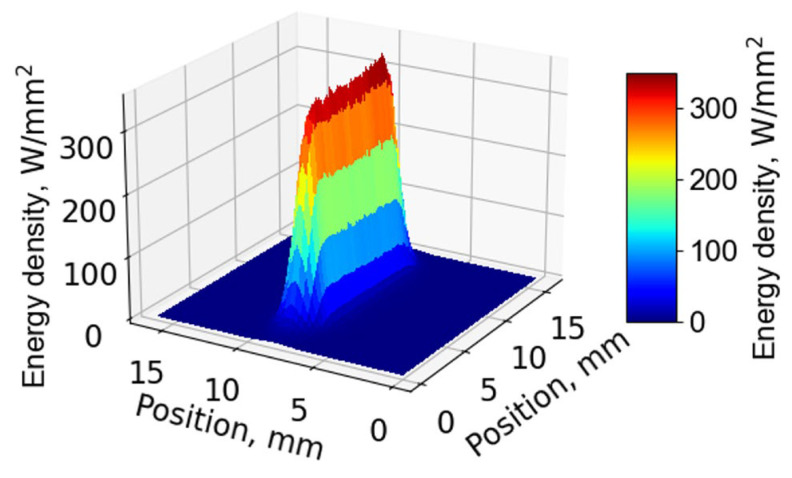
Energy distribution of laser beam spot.

**Figure 4:**
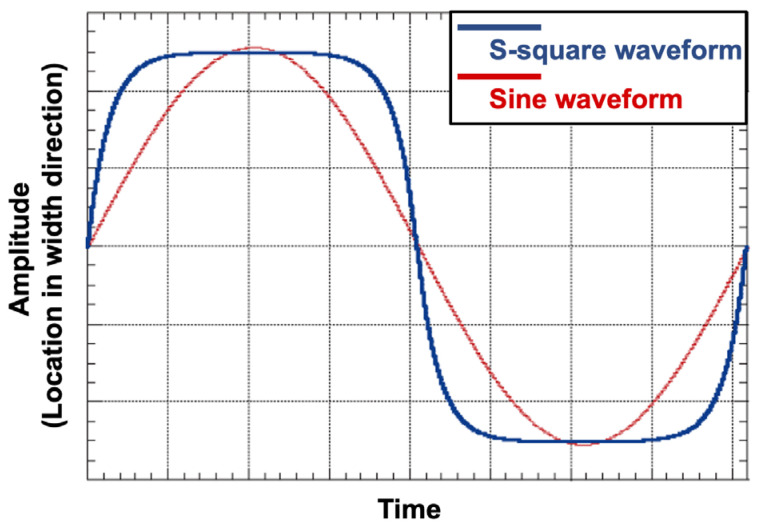
Weaving motion of laser beam spot.

The length of the laser spot was oriented parallel to the welding direction. Energy density distribution of the laser beam is shown in Figure 3. In addition, a small laser beam with oscillatory motion was used for reaching from side to side of the narrow joint. The smoothing-square (s-square) oscillation waveform is shown in Figure 4. The time duration at high and low amplitudes (beam located at groove wall sides) was longer than that of a sine waveform. This characteristic assisted in suppression of the incomplete fusion that usually occurs at the groove walls of a narrow joint.

Experiments were conducted under the conditions shown in Table 2 to investigate the effect of heat input by changing the welding speed from 0.3 m/min to 0.75 m/min on the occurrence of defects, hardness distribution, and grain size of the HAZ. The wire feeding speed was varied following welding speed to maintain the deposition rate per unit length. A diode laser oscillator was used as the main heat source, and the laser power was fixed at 6 kW. A 10 mm thick groove could be welded in one pass under these conditions. The hot-wire current was set to a suitable limiting current value for each wire feeding speed at which no fusing or fluctuation occurred during welding under the 80 mm energization distance for joule heating.

The welded joints were cut, and the length of the lack-of-fusion region was measured from three cross sections and represented as the lack-of-fusion ratio relative to the total groove length. Vickers hardness was measured in the middle of the joint thickness on the bead width direction.

**Figure 5:**
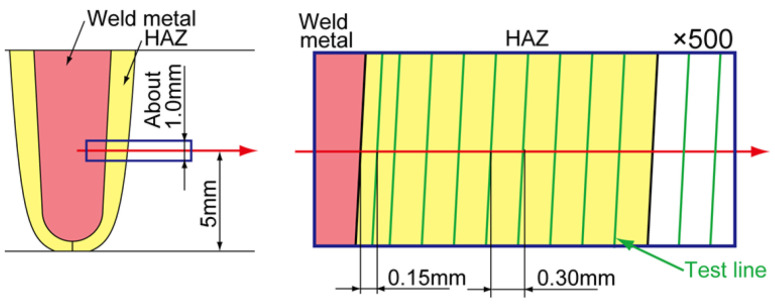
Welding and evaluation region.

The grain size was measured using an optical microscope and linear-intercept method. The measurement locations and methods are shown in Figure 5. The fine-grained region was not measured due to the difficulty of counting intercepts for the high-density grains.

The heat input of this welding method was estimated using the measured grain size by performing an inverse estimation using the grain growth equation from Ikawa et al. [25]. The grain growth equation for high-tensile steel is expressed as

(1) $$\begin{array}{c} \log_{10}\left(D^{4}-D_{0}^{4}\right)=-92.64+2\log_{10}\eta^{\prime }q^{\prime }+\frac{1.291\times 10^{-1}}{\left(\frac{l^{\prime }}{\eta^{\prime }q^{\prime }}\right)+1.587\times 10^{-3}}, \end{array}$$

where *D* is the grain diameter after welding, *D*_0_ is the grain diameter of the base metal (27.3 μm), *η*′*q*′ is the amount of heat input, and *l*′ is the distance from the weld.

**Figure 6:**
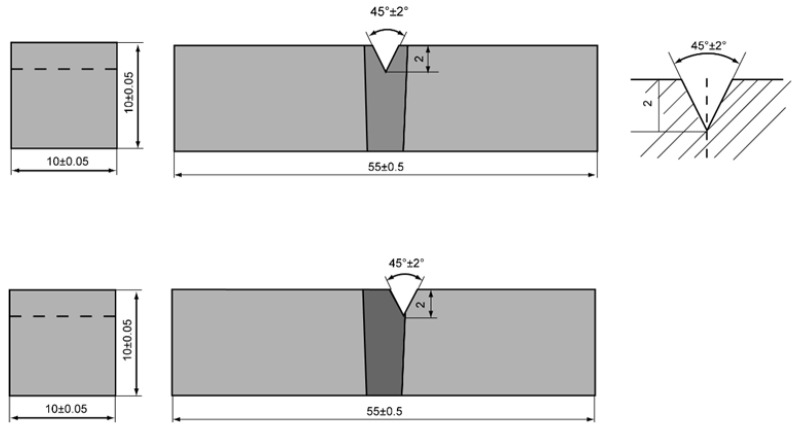
Charpy impact testing samples (unit: mm).

On the basis of the results of the varied welding speed experiments, the welding conditions were optimized for a test specimen with a plate thickness of 12 mm, and a Charpy impact test and tensile test were conducted. The welding conditions are shown in Table 3. The Charpy impact test was conducted using a V-notch test specimen at temperatures of −40 °C and −20 °C. In addition, experiments were conducted at two positions for the V-notch: the weld metal and fusion boundary. The dimensions of the test specimens and test positions are shown in Figure 6.

**Figure 7:**
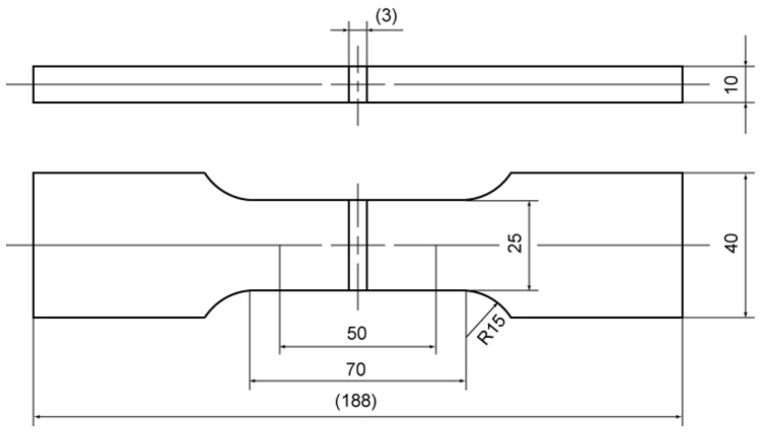
Tensile test specimen (unit: mm).

The tensile test was conducted in accordance with the JIS standard [26], and the dimensions used were those of specimen No. 5, as specified in the JIS standard [26]. The shape of the tensile specimen is shown in Figure 7.

### 2.2. Finite Element Analysis Model

**Figure 8:**
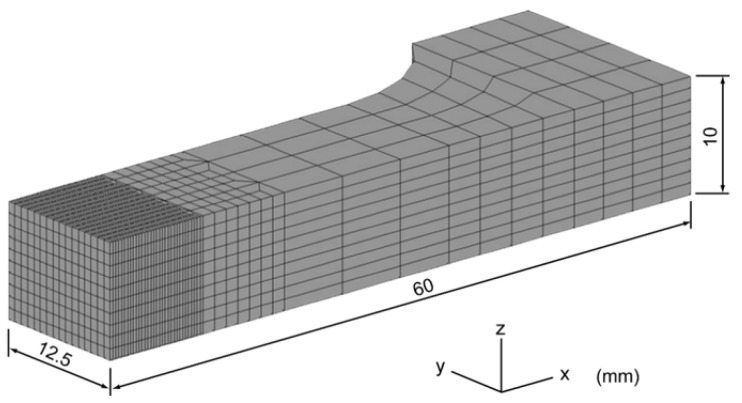
Finite element model grid.

**Figure 9:**
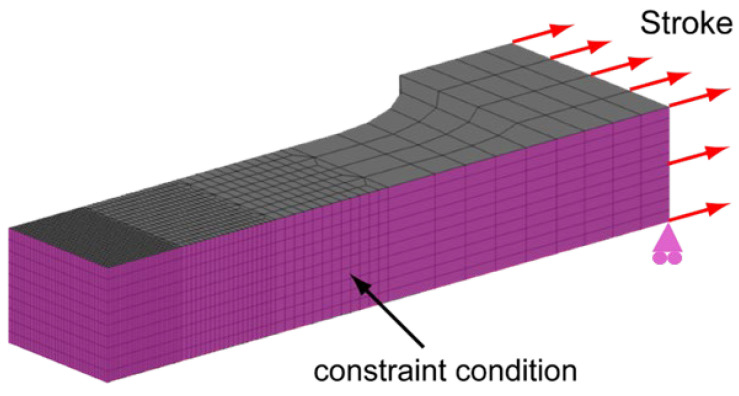
Boundary conditions and constraints of finite element model.

To clarify the effect of the width of the undermatched zone of the weld metal and softening HAZ zone, FEM analyses were conducted using tensile test models. The analysis was conducted on a one-quarter model using MARC. The region included the weld metal, softened zone of the HAZ, and base metal. The analysis model is shown in Figure 8. The boundary conditions were set to be symmetrical on the symmetry plane, and a forced variation of 0.0415 mm per step was applied in the tensile direction. The analysis was stopped when the maximum contact variation in the *X*-axis (force direction) reached 3 mm. The boundary conditions are shown in Figure 9.

**Figure 10:**
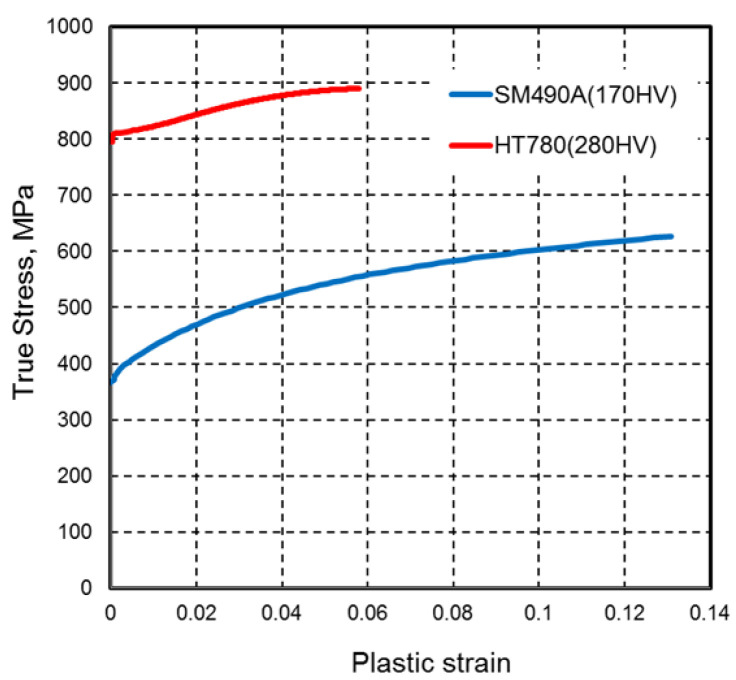
True strain–stress relation of 780 MPa-class and 490 MPa-class high-tensile strength steels.

**Figure 11:**
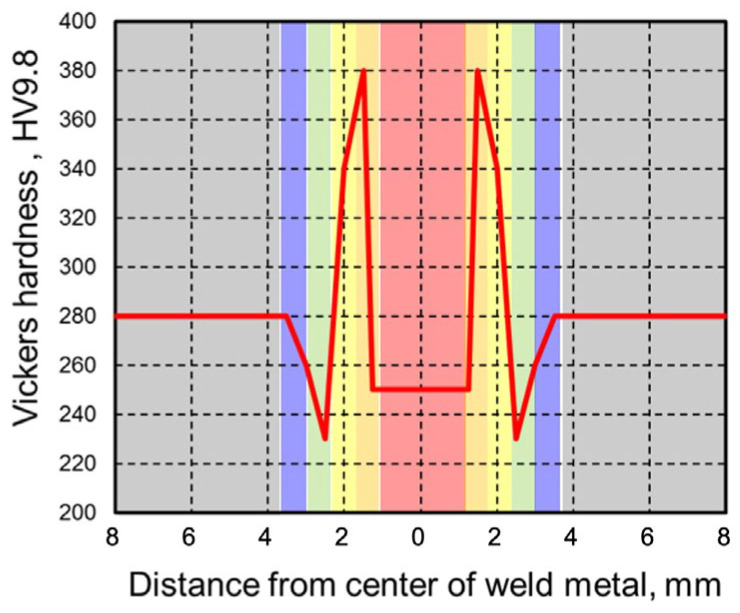
Example of hardness distribution of finite element model (red: weld metal, dark yellow~light yellow: hardened HAZ, green~blue: softened HAZ, gray: base metal).

On the basis of hardness measurements of actual welded joints, the changes in mechanical properties were estimated to simulate a joint where the hardness changed between the weld metal, HAZ softening area, and base metal. Specifically, the mechanical properties were simulated by performing linear interpolation at each hardness level [27] based on the true stress–true strain relationships of 780 MPa-class high-tensile strength steel (base metal hardness: 280 HV) which is same as the used base material and 490 MPa-class high-tensile strength steel (base metal hardness: 170 HV) which is same as the used filler metal. The true stress–true strain relationships used for interpolation are shown in Figure 10. The hardened and softened zones were divided into regions of 0.5 mm each. An example of the hardness distribution set in the analysis model is shown in Figure 11.

The effect of weld metal width on the joint strength was analyzed by changing the weld metal width, which was considered when using low-strength wire. The hardness of the weld metal was set at 220 HV, which is softer than the base metal hardness of 280 HV. The width of the weld metal was set at three conditions of 2.5, 5.0, and 7.5 mm. The analysis conditions near the weld metal are shown in Table 4.

The softened HAZ width was varied in an even-matched joint where the hardness of the weld metal was equivalent to the base metal hardness of 280 HV. The analysis was performed under three conditions with softened HAZ widths in region 3 of 1.0, 1.5, and 2.0 mm. The analysis conditions are shown in Table 5.

## 3. Results and Discussion

### 3.1. Effect of Welding Speed on Microstructure and Hardness Distribution

**Figure 12:**
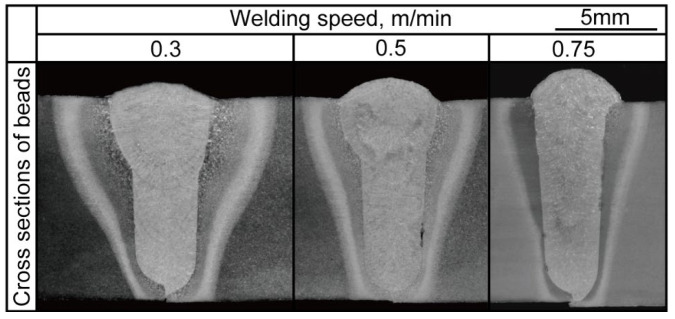
Cross sections under three welding speed conditions.

The experimental conditions are detailed in Table 2. Cross-sectional photographs of the welded joints are presented in Figure 12. Qualitative observation indicates that the welded area and mid-bottom bead width across all conditions showed insignificant variation with changes in travel speed. The mid-bottom bead width was influenced by the welding speed, which aligns with findings from previous studies [28,29]. The top width of the welded joint showed slight reductions at travel speeds of 0.5 m/min compared with 0.3 m/min, while a more significant reduction was observed at 0.75 m/min due to the lower heat input under the higher welding speed condition [30].

**Figure 13:**
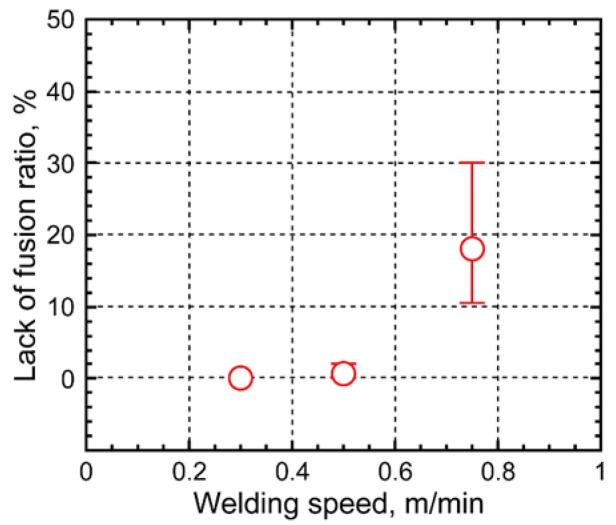
Lack-of-fusion ratios as a function of welding speed.

As shown in the cross section in Figure 12, a lack of fusion at the groove wall could be observed for travel speed conditions of 0.5 m/min and 0.75 m/min. This lack of fusion occurred due to insufficient heat input to balance the deposition rate, laser energy density, and welding speed [30,31]. The lack-of-fusion ratio at each welding speed was measured from the cross-sectional observations and is shown in Figure 13. No lack of fusion was observed at a welding speed of 0.3 m/min.

**Figure 14:**
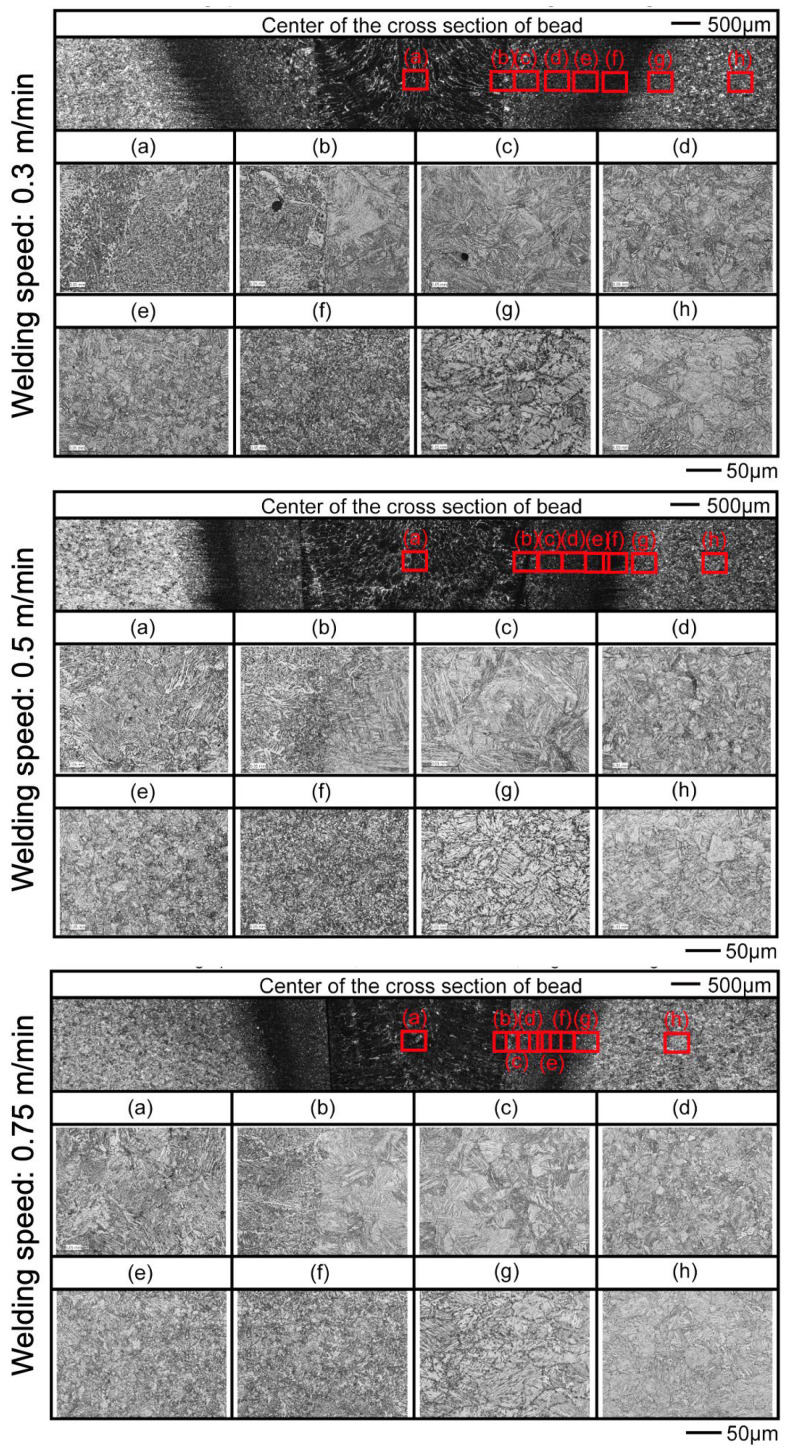
Microstructures of joints welded at various welding speed conditions.

The microstructure was observed at the center of the plate thickness (5 mm from the bottom of the joint) on cross-sectional samples. Images obtained using an optical microscope are presented in Figure 14. The macroscopic images indicate that the HAZ width decreased with increasing welding speed, as confirmed in both Figure 12 and Figure 14. The microstructure of the base metal, shown in location (h) for all conditions, exhibited a morphology characterized by gray polygonal grains with dark black stripes, which several studies have identified as tempered martensite [22,32,33,34]. Additionally, the presence of thin white elongated grains indicated the presence of bainite and ferrite [33,34]. In the welded metal at location (a) with a travel speed of 0.3 m/min, the microstructure revealed acicular ferrite and grain boundary ferrite (GBF) [33]. However, as the travel speed increased to 0.5 and 0.75 m/min, the microstructure showed a higher fraction of bainite and acicular ferrite, with a lower fraction of GBF [34]. Similar morphologies were observed at all travel speed conditions across the fusion line, HAZ, and base metal, although the width of this area decreased due to the reduction of heat input with increasing travel speed.

A large fraction of bainite was observed close to the fusion line at location (b), extending from location (c) to location (e), where the size of the bainite decreased. This behavior has been attributed to grain growth resulting from heat exposure, as reported in previous studies [34]. Small equiaxed grains were found at location (f), suggesting recrystallization of the metal [34]. Additionally, nucleation was observed at the grain boundaries at location (g), with high carbon diffusion occurring at these boundaries, for which similar behavior has been documented [33]. These results indicate that the material subjected to heat during the welding process consisted of recrystallized regions and areas of grain growth, suggesting that this region may have contributed to softening in the HAZ. Furthermore, the literature reports similar microstructural behavior and morphology, revealing both hardening and softening zones in the HAZ of welded joints. This variation in microstructure results in higher mechanical properties in these regions compared with those of the base metal [35,36]. However, further investigations are needed to fully understand the characteristics of high-strength steel joints.

**Figure 15:**
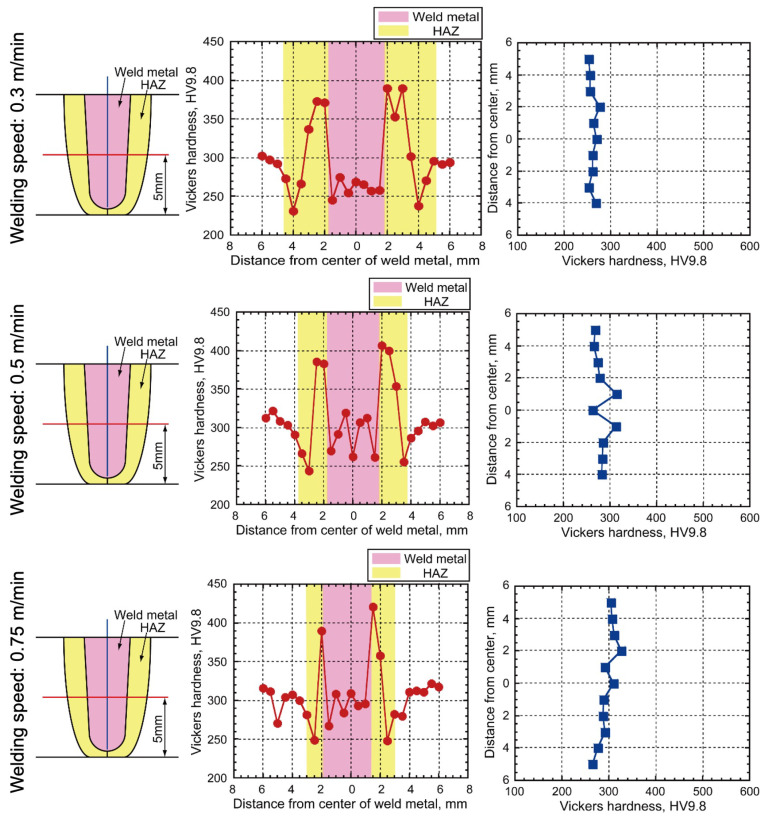
Vickers hardness distribution results.

The results of Vickers hardness measurements at the midpoint of the welded joint are shown in Figure 15. The hardness distribution exhibited a similar profile along the welded joint across all conditions, with increased hardness influenced by the refined grain structure during recrystallization at location (f) in Figure 14. The reduction in hardness within the HAZ was confirmed by the microstructure observed at location (g) in Figure 14. This hardness distribution result aligns with findings from several studies on high-strength steel welding [22,32].

Welding speeds of 0.3, 0.5, and 0.75 m/min resulted in hardness values of the welded metal of about 260, 300, and 300 HV, respectively. The hardness increase resulted from suppression of GBF at the 0.5 and 0.75 m/min travel speed conditions. Furthermore, the average hardness of the welded metal at the welding speed of 0.3 m/min was undermatched to base-metal hardness in this study.

**Figure 16:**
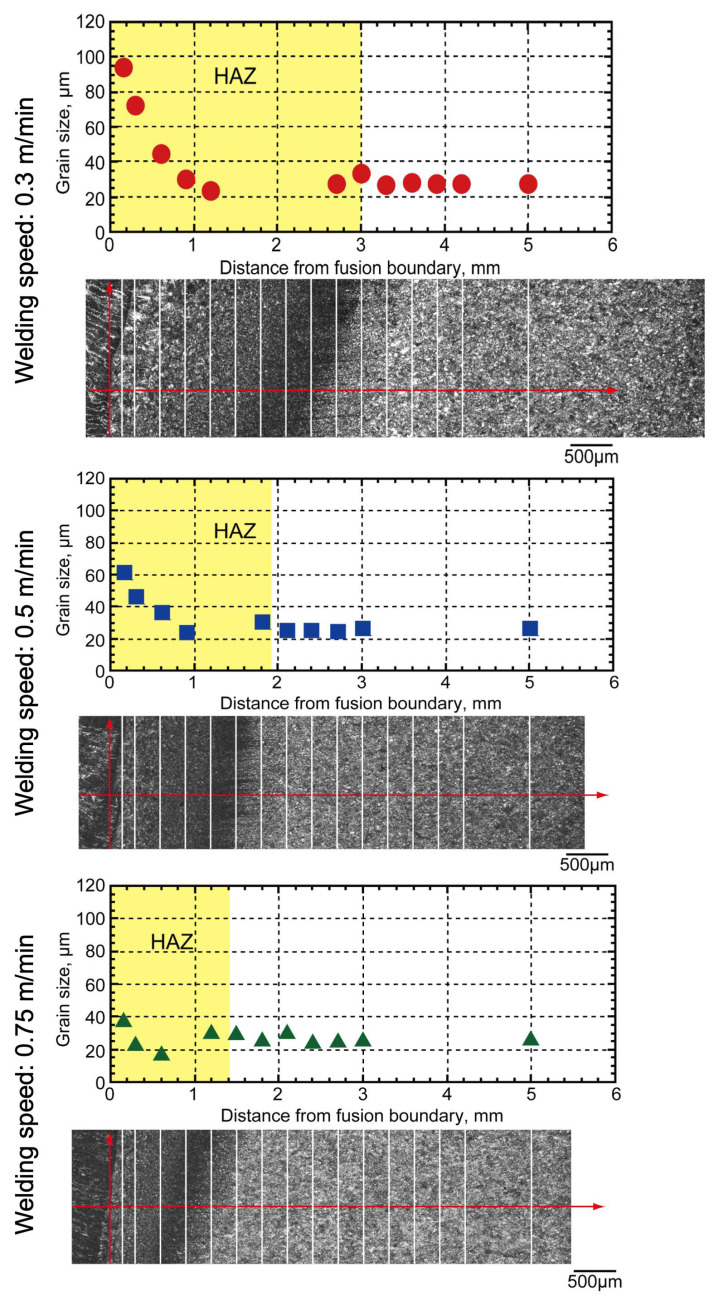
Measured grain size from fusion boundary.

The grain size measurements are presented in Figure 16. The grain size decreased from the fusion line to the base metal in all conditions [37,38]. As the travel speed increased, the grain size reduced due to the influence of lower heat input and shortening of the HAZ region [38]. Moreover, the region near the fusion boundary revealed large grain sizes of 100, 60, and 40 μm for travel speeds of 0.3, 0.45, and 0.75 m/min, respectively. In contrast, grain sizes in the base metal were smaller, indicating that this region can be defined as a coarsening area resulting from heat exposure during welding, which promotes grain growth [37,38].

**Figure 17:**
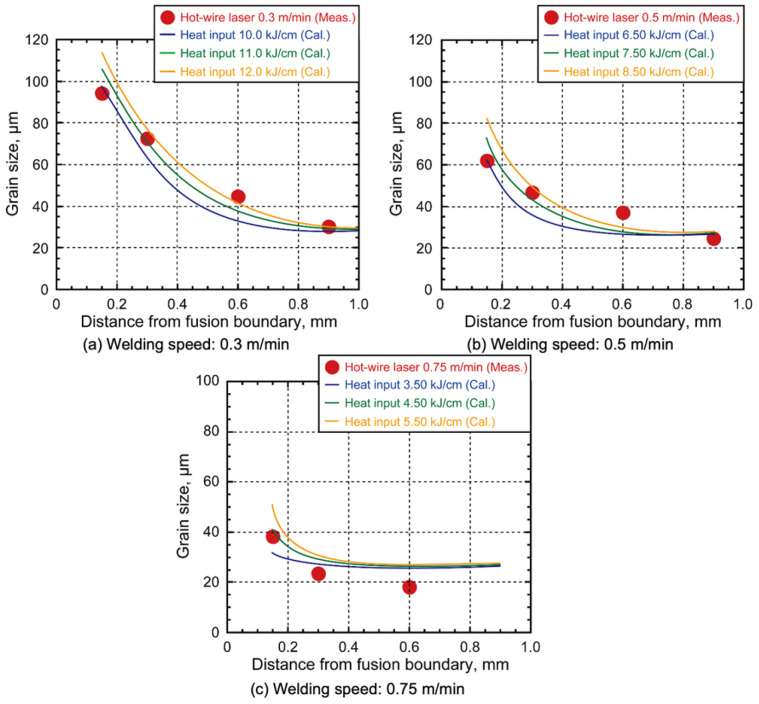
Comparison of calculation heat input and measured grain sizes.

The width of the grain-coarsening region was found to be proportional to the heat input. On the basis of these observations, the heat input was estimated using Equation (1). The heat input was calculated using the measured grain size results from Figure 16, taken at a distance of 0.15 mm from the fusion boundary [25], across all travel speed conditions, and substituted into Equation (1). The results of these calculations are shown in Figure 17. Comparison of the calculated values shows that the heat inputs were approximately 11.0 kJ/cm, 7.5 kJ/cm, and 4.5 kJ/cm at welding speeds of 0.3 m/min, 0.5 m/min, and 0.75 m/min, respectively, as indicated by the best fit to the experimental grain size results.

This hot-wire laser narrow-gap welding process is therefore considered a low-heat input welding method compared with conventional processes such as GMAW [39,40,41,42] or laser welding [43,44].

### 3.2. Joint Evaluation of Mechanical Properties and Finite Element Model Analysis

**Figure 18:**
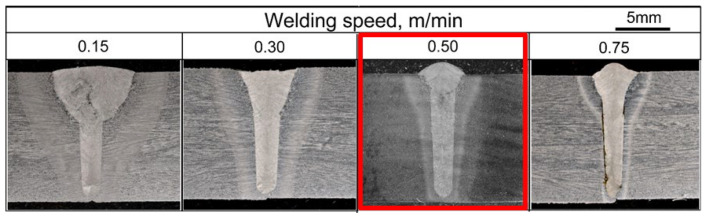
Cross-section appearances under various welding speed conditions (red: selected optimum welding speed).

Experiments were conducted at various welding speeds on material of 12 mm thickness to evaluate the mechanical properties. The conditions are listed in Table 3. Cross-sectional observation of bead formation of a butt joint is shown in Figure 18. The joint welded at a speed of 0.5 m/min was selected as the optimized condition due to being the highest speed at which uniform penetration with defect-free formation was achieved. Therefore, the condition of 0.5 m/min was selected for testing of mechanical properties, including Chappy impact and tensile tests.

**Figure 19:**
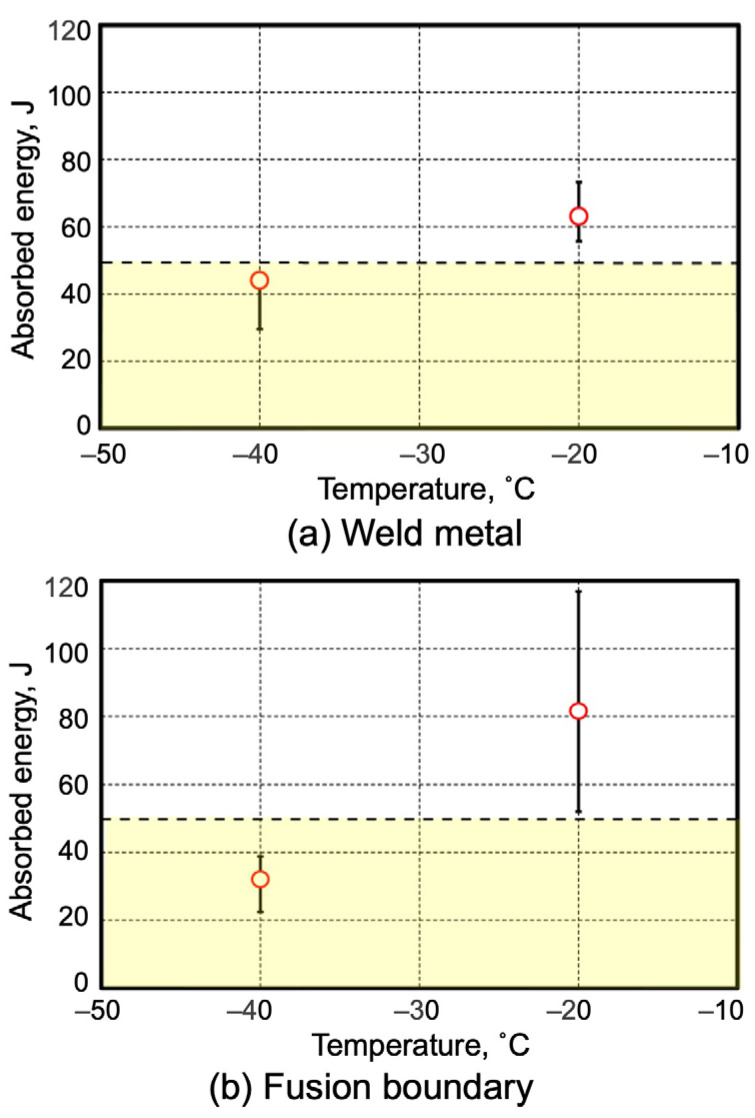
Charpy impact test results.

The Charpy impact test was carried out at temperatures of −20 °C and −40 °C at the weld metal center and fusion boundary. The results are represented in Figure 19. At the positions of weld metal (a) and fusion boundary (b) during −20 °C testing, the result of 47 J satisfies the requirement for absorption energy higher than that of the joint, which is influenced by the microstructure [22,45] of a joint similar to that shown in Figure 14. Remark occurred at the fusion boundary due to higher absorption energy compared with that of the weld metal due to formation of a large tempered structure from heat subjection during welding [32]. Moreover, this area was exposed to large fracture path deviation (FPD) of the testing sample, evaluated close to the boundary of the weld metal and base metal areas. During testing at −40 °C, the joint energy absorption was shown to be below the designed value of 47 J for both the weld metal (a) and fusion boundary (b) areas. These were influenced by the microstructure comprising the larger bainite fraction in weld metal and temper microstructure near fusion boundary during this low-temperature testing [45]. However, selection of a filler wire with higher toughness or a post-heat treatment process might be considered to modify the microstructure in this weld metal area for better energy absorption [45].

**Figure 20:**
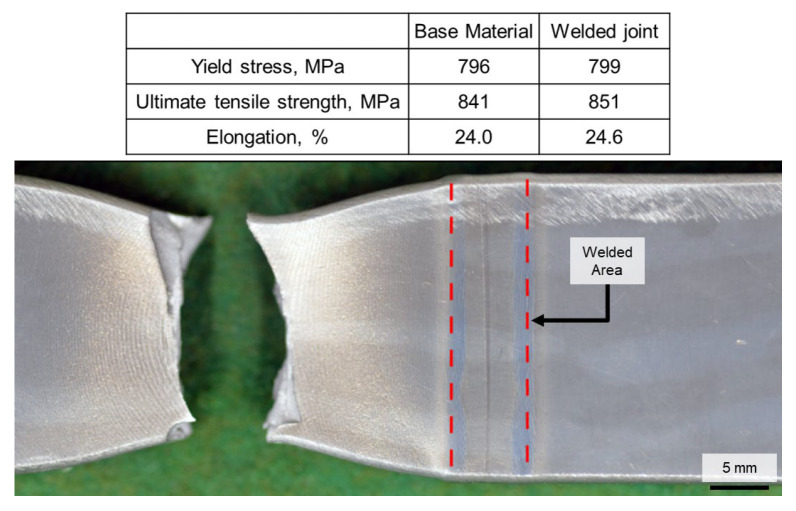
Tensile test result on welded joint (red dashed lines: fusion boundaries).

Tensile testing was conducted on samples prepared from the base material and welded joint. The yield stress, ultimate tensile strength, and elongation were obtained. The welded joint was found to be fractured in the base metal area, while the overall welded joint strength was not affected by the hot-wire laser welding process. The appearance of the welded joint tensile sample after the testing is shown in Figure 20.

Results from evaluations of several metallurgy and mechanical properties suggested and confirmed that the welded joint exhibited a softening region in the HAZ, with the welded metal being softer than the base metal [22,33]. However, during tensile testing, failure of the welded joint was observed to occur in the base metal. This behavior, as noted in [46], is influenced by the narrow undermatched region of the welded joint (either in the welded metal or HAZ), which does not undervalue the overall deterioration of joint properties (tensile strength), resulting in joint fractures occurring in the base material region.

### 3.3. Finite Element Model Analysis Results

**Figure 21:**
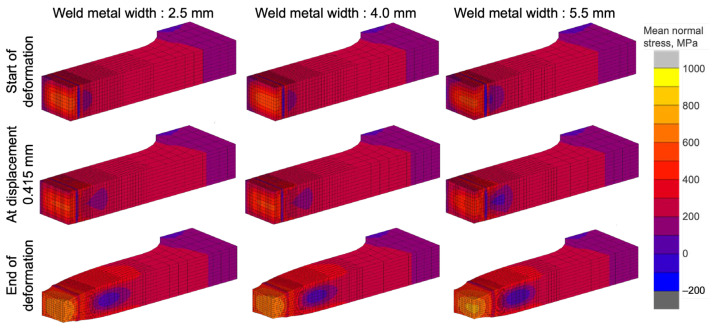
Mean nominal stress contours and deformation in analysis of undermatched weld metal width effect.

The effect of the weld metal width on the joint strength was analyzed for various weld metal widths using FEM models in which the welded joint was considered when low-strength wire was used (i.e., undermatched joints). Contour diagrams of the mean normal stress just after yielding (start of plastic deformation), during progression of plastic deformation (displacement at 0.415 mm), and at the end of the analysis (displacement at 3.00 mm) for each condition are shown in Table 4 and Figure 21. At the start of plastic deformation, the stress in the narrow range of the HAZ hardening zone was low, while that in the surrounding weld metal and HAZ softening zone was very high. As deformation progressed (displacement at 0.415 mm), this trend became more pronounced. At the end of the analysis, when high stress conditions prevail, displacement and necking were observed on the weld metal located at center of the specimen (left edge of the analysis model).

**Figure 22:**
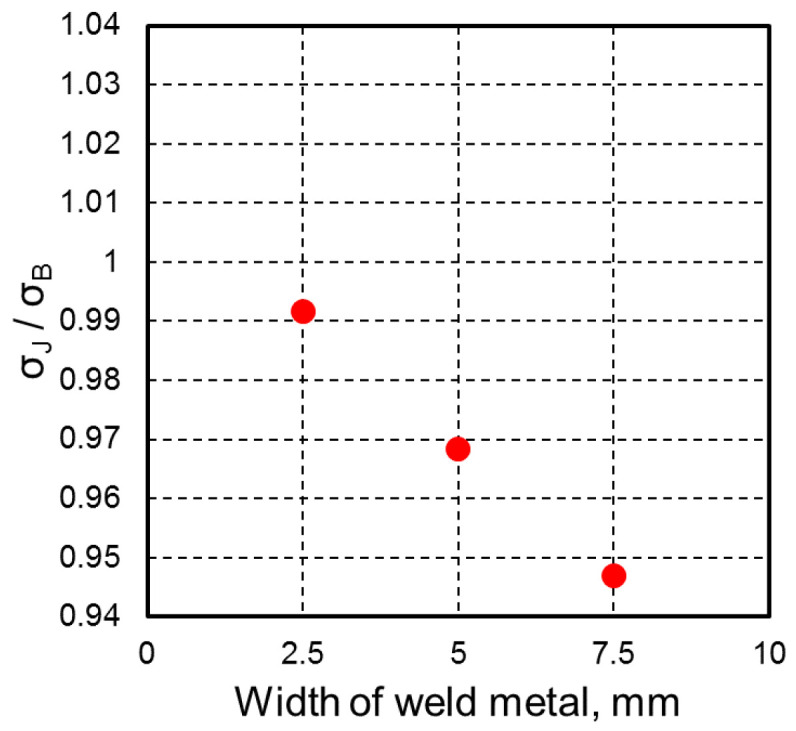
Effect of weld metal width on joint efficiency (σ_J_/σ_B_).

The joint nominal tensile strength (σ_J_) obtained from FEM analysis was compared by normalization using the base material tensile strength (σ_B_). The result is represented as joint efficiency in Figure 22. Although the welded metal width increased, the efficiency of the welded joint decreased, as reported by several other researchers who used FEM analyses [47,48]. However, the highest joint efficiency could be achieved at the narrowest welded width of 2.5 mm, with retention of more than 99% tensile strength. The FEM result indicated that welded joints could be obtained with strength that was comparable to that of the base material with a narrow softening region, even when using an undermatched filler wire. However, as shown by the result in Figure 21, the softer weld metal joint failed at the weld metal region, raising concerns about a decrease in fracture elongation and other properties.

**Figure 23:**
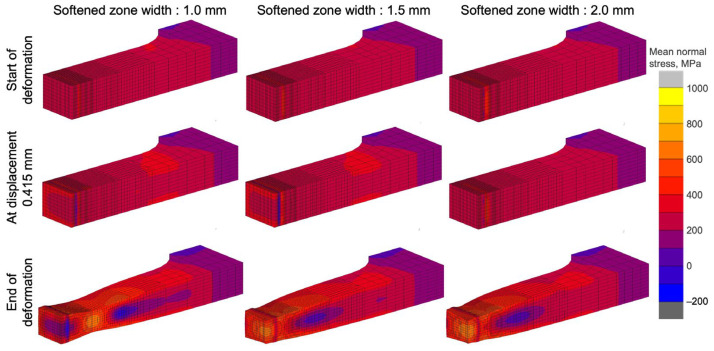
Mean nominal stress contours and deformation in analysis of softened heat-affected zone width effect.

To confirm the effect of the softened HAZ width on joint strength, analyses were carried out at three positions of the softened zone width in an even-matched joint, which is defined as constrained by equivalent hardnesses of the weld metal and base metal. The analyses were carried out on the basis of the conditions shown in Table 5. Contour diagrams of the mean normal stress at the time of yielding, at the start of plastic deformation (displacement: 0.415 mm), and at the end of deformation are shown in Figure 23. At the start of deformation, there was no stress concentration in the weld metal and HAZ regions, and similarity in all conditions. Deformation progress to softened zone widths of 1.0 mm and 2.0 mm clearly showed a low-stress (blue) region adjacent to the weld metal, which is a high-hardness region. At the end of deformation, the 1.0 mm softened zone width showed large deformation and thickness decrease in the base metal, and the highest stress and necking deformation occurred approximately 10 mm from the center of the weld metal. In contrast, 1.5 mm and 2.0 mm softened zone widths showed the highest stress and necking deformation at the softened HAZ region.

**Figure 24:**
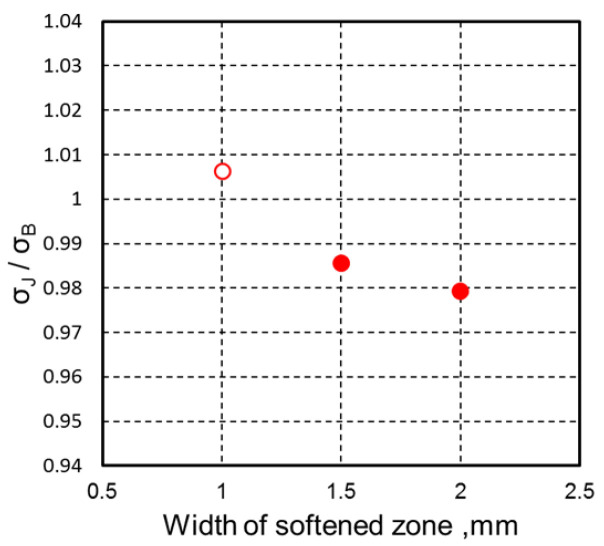
Effect of softened heat-affected zone width on joint efficiency (σ_J_/σ_B_) (red dot: < 1.0, red circle: > 1.0).

The effect of joint efficiency on softened zone width was summarized by considering the nominal stress of the joint (σ_J_) during FEM analysis of tensile strength. The result was calculated with nominal tensile stress of the base material (σ_B_) and is represented in Figure 24. The results indicate that with the narrowest softened zone (1.0 mm), the joint efficiency reached 100% compared with that of the base material. In contrast, a wider softened zone resulted in lower joint efficiency due to plastic deformation in the softened region, leading to lower strength. On the basis of these findings, several articles [47,48,49] have reported that an increase in strength and decrease in the width of the softened HAZ can enhance the strength of the butt joints: effects of the strength and width of a hardened HAZ on butt joint strength were insignificant.

Failure locations can occur in either the weld metal or base metal, depending on the mismatch ratio and width of the softened zone. Mechanical behavior of the overall joint strength is influenced by the plastic distribution within the softened HAZ. Narrow softened regions can achieve strengths comparable to those of the welded metal or base metal due to the constraints imposed by the adjacent unyielded materials. In cases of mismatched joints, a decrease in joint ductility is observed, which varies based on the failure location and hardening properties in the failure zone [49]. However, on the basis of our FEM results, achieving a highly efficient joint requires a strategy to narrow the HAZ soft zone as much as possible. This approach minimizes plastic deformation in the softened region, thereby reducing deformation and stress on the base metal and ensuring that the ductility remains comparable to that of the base metal.

## 4. Conclusions

The effect of welding speed on the microstructure and hardness of a welded joint was investigated under conditions of welding 10 mm thick 780 MPa-class high-tensile strength steel in a single pass using hot-wire laser narrow-gap welding. The heat input was estimated from the measured grain size in the HAZ. In addition, on the basis of the experimental results, a strength-evaluation specimen was welded under optimized welding conditions. The effects of undermatched weld metal width and softened HAZ width on the joint strength were clarified using FEM analysis. The conclusions obtained in this study are as follows.

1. Welding at various speeds of 0.3, 0.5, and 0.75 m/min, and hot-wire narrow-gap laser welding was performed in a single pass using 490 MPa-class wire as the filler wire and 10 mm thick 780 MPa-class high-tensile steel. The lack-of-fusion defect ratio was minimized at welding speeds of 0.3 and 0.5 m/min, and no other defects occurred. There was a difference in the hardness of the weld metal, and the weld speed of 0.3 m/min resulted in an undermatched joint due to a slower cooling speed compared with those welded at 0.5 and 0.75 m/min.
2. The HAZ structures were observed. Grain sizes in the coarsening region near the weld metal were approximately 100 μm, 60 μm, and 40 μm at welding speeds of 0.3 m/min, 0.5 m/min, and 0.75 m/min, respectively. On the basis of these results, the respective heat inputs were estimated as 11.0 kJ/cm, 7.5 kJ/cm, and 4.5 kJ/cm. The estimated heat inputs are considered significantly low compared with those of other welding methods. This confirmed that a low-heat input hot-wire laser welding process had been carried out.
3. Charpy impact and tensile tests were carried out on joints that had been welded under optimized conditions. Charpy impact tests were carried out on the weld metal and at the fusion boundary. It was confirmed that the absorbed energy was 47 J or more at all positions when the test temperature was −20 °C. The location of the tensile test failure in the base metal confirmed that it was possible to obtain sound strength, compared with that of the base metal, even if softening occurred in the HAZ.
4. FEM analysis was carried out for various widths of the softened area to confirm the effect of the weld metal on the strength of an undermatched joint. When the width of the weld metal was limited to 2.5 mm, the joint efficiency was 99% or more and, even when a soft weld metal area was produced, it was possible to obtain a tensile strength almost the same as that of the base material.
5. FEM analysis was carried out to confirm the effect of changes in the HAZ softening region width on the strength of the joint. It was confirmed that the joint efficiency reached 100% when the softened HAZ width was very narrow (1.0 mm in this study), and fracture occurred in the base metal.
6. Use of the narrow-gap hot-wire laser welding method enabled production of sound joints with a high balance between strength and toughness by the suppression of HAZ softening using a low-heat input process and the application of soft wire.

In the next study, investigations of other kinds of base materials and filler wires to achieve sound joints with higher balance of some properties will be performed. In addition, the hot-wire laser welding process will be applied to enable more efficient welding for thicker plates with sufficient joint properties.

## Figures and Tables

**Table 1:** Chemical compositions of filler wire and base metal.

Material	Chemical Composition, Mass%									
	C	Si	Mn	P	S	Cu	Cr	Nb	V	Ti
Base metal 780 MPa-class steel	0.14	0.25	1.32	0.011	0.004	0.01	0.25	0.002	0.002	0.01
Filler wire JIS Z3316W49A3U16	0.06	0.73	1.74	0.021	0.007	0.24	0.03	--	--	--

**Table 2:** Experimental conditions.

Parameter	(a)	(b)	(c)
Laser power, kW	6.0		
Laser spot, mm	1.6 × 11.0		
Laser irradiation angle, deg	5		
Defocus length, mm	10		
Welding speed, m/min	0.3	0.5	0.75
Energization distance, mm	80		
Wire feeding speed, m/min	8.2	13.7	20.5
Hot-wire current, A	158	178	248
Weaving frequency, Hz	10		

**Table 3:** Welding conditions for Charpy impact test and tensile test.

Parameter	
Laser power, kW	6.0
Laser spot, mm	1.6 × 11.0
Laser irradiation angle, deg	5
Defocus length, mm	0
Welding speed, m/min	0.5
Energization distance, mm	80
Wire feeding speed, m/min	12.7
Hot-wire current, A	180
Weaving frequency, Hz	15

**Table 4:** Finite element analysis conditions for undermatched weld metal width effect.

Region	Vickers Hardness, HV	Width of Each Region (Half Width), mm		
Weld metal	220	2.5 (1.25)	5.0 (2.5)	7.5 (3.75)
HAZ 1	380	0.5		
HAZ 2	340	0.5		
HAZ 3	230	0.5		
HAZ 4	260	0.5		
Base metal	280	--		

**Table 5:** Finite element model conditions for softened heat-affected zone width effect.

Region	Vickers Hardness, HV	Width of Each Region (Half Width), mm		
Weld metal	280	2.5 (1.25)		
HAZ 1	380	0.5		
HAZ 2	340	0.5		
HAZ 3	230	0.5	0.75	1.0
HAZ 4	260	0.5		
Base metal	280	--		

## Data Availability

The original contributions presented in the study are included in the article; further inquiries can be directed to the corresponding author.
